# Soil Pollution and Its Interrelation with Interfacial Chemistry

**DOI:** 10.3390/molecules30122636

**Published:** 2025-06-18

**Authors:** Patricia Omo-Okoro, Peter Ofori, Vijitha Amalapridman, Arezoo Dadrasnia, Lord Abbey, Chijioke Emenike

**Affiliations:** 1 Department of Plant, Food and Environmental Sciences, Faculty of Agriculture, Dalhousie University, Truro, NS B2N 5E3, Canada; patricia.omo-okoro@dal.ca (P.O.-O.); pt269228@dal.ca (P.O.); vj834155@dal.ca (V.A.); labbey@dal.ca (L.A.); 2 Biotechnology Centre, College of Basic and Applied Sciences, University of Ghana, Legon, Accra P.O. Box LG 1195, Ghana; 3 Department of Biosystems Technology, Faculty of Technology, University of Jaffna, Kilinochchi 42400, Sri Lanka; 4 BETA Tech Center, University of Vic-Central University of Catalonia, C.de La Laura 13, 08500 Vic, Spain; are.dadrasnia@gmail.com; 5 Natural and Applied Sciences, Hezekiah University, Nkwerre 47119, Nigeria

**Keywords:** soil pollution, interfacial chemistry, soil treatment, contaminant, interfaces, remediation

## Abstract

This review offers an in-depth analysis of soil contamination, discussing the origins, impacts, and remediation strategies, as well as the complex connections with interfacial chemistry. Interfacial chemistry plays a critical role in addressing soil contamination by governing the interactions between pollutants, soil particles, water, and remediation agents at phase boundaries (solid–liquid, solid–gas). Some key aspects include adsorption/desorption that controls pollutants binding to soil surfaces; chemical transformation which facilitates redox, hydrolysis, or catalytic reactions at interfaces to degrade contaminants; colloidal transport that affects the movement of nanoparticle-bound contaminants through soil pores; and techniques like soil washing, phytoremediation and permeable reactive barriers that can neutralize soil pollutants. The combination of interfacial chemistry and soil remediation techniques offers rich opportunities for improving predictive models of contaminant fate. Such approaches represent a paradigm shift from equilibrium-based remediation to dynamic process management. The review demonstrates how heterogeneous interfaces and molecular-scale dynamics dictate contaminant behavior. Furthermore, in addition to consolidating existing knowledge, the review also pioneers new directions by revealing how interfacial processes can optimize soil decontamination, offering actionable insights for researchers and policy makers. By understanding and manipulating interfacial chemical processes, scientists can develop more precise and sustainable cleanup methods.

## 1. Introduction

Contaminated soil presents major risks to human health, disrupts natural habitats, and endangers the reliability of our food systems [1]. In recent decades, it has become one of the foremost environmental concerns, impacting biodiversity, agricultural yields, and human well-being. The deterioration of soil quality not only reduces the diversity of living organisms but also undermines vital soil functions such as water purification, nutrient recycling, and carbon storage [2]. Soil pollution arises from diverse anthropogenic and natural sources, with profound implications for ecosystem stability and human health. Industrialization remains a dominant contributor, introducing hazardous elements such as lead (Pb), cadmium (Cd), and arsenic (As) through mining, manufacturing, and improper waste disposal [3]. Agricultural intensification further exacerbates the problem, as excessive pesticide use, synthetic fertilizers, and irrigation with contaminated water introduce persistent organic pollutants (POPs) and nutrient imbalances into soils [4]. Urban expansion and infrastructure development contribute through construction waste, hydrocarbon spills, and atmospheric deposition of particulate matter [5]. While natural processes such as mineral erosion and volcanic activity contribute to soil pollution, human activities such as industrial operations, intensive farming, and improper waste disposal are the primary drivers of contamination [6].

Interfacial chemical processes that take place at the interfaces between several soil phases primarily control the fate, behavior, and removal of these pollutants. These interfaces serve as crucial areas where molecular interactions determine whether contaminants are released into groundwater systems or stay trapped in the soil matrix. Adsorption–desorption phenomena, surface precipitation, ion exchange reactions, and redox transformations are important interfacial processes. The physicochemical characteristics of the soil, including its pH, redox potential, organic matter content, and mineralogical composition, further impact the intricacy of these interactions.

The ramifications of soil pollution are far-reaching. Microbial communities, which drive essential nutrient cycles, are particularly vulnerable to pollutant toxicity, leading to reduced enzymatic activity and impaired organic matter decomposition [7]. Such disruptions flow through food webs, affecting soil invertebrates, plant health, and ultimately higher trophic levels [8]. Heavy metals like mercury (Hg) and chromium (Cr) bioaccumulate in crops may enter human diets and potentially cause chronic health disorders, including neurological damage and carcinogenic effects [9]. From a socioeconomic perspective, soil degradation imposes substantial costs through reduced agricultural yields, water treatment expenses, and healthcare burdens associated with pollution-related diseases [10]. Developing nations face disproportionate impacts due to weaker regulatory frameworks and reliance on subsistence farming, where contaminated soils directly threaten livelihoods [11].

The persistence of contaminants in the soil matrix disrupts soil ecosystems and poses long-term risks to food security and public health, necessitating effective remediation strategies. The spectrum of soil contaminants includes heavy metals such as lead, cadmium, arsenic and others, POPs including polycyclic aromatic hydrocarbons (PAHs), polychlorinated biphenyls (PCBs), pesticides, petroleum hydrocarbons, and emerging contaminants of concern, such as pharmaceuticals and microplastics. These pollutants pose substantial risks to ecosystem functioning, food security, and human health through various exposure pathways. Effective remediation of contaminated soil requires understanding contaminant–soil interactions governed by interfacial chemistry principles [12].

The interrelation between soil pollution and interfacial chemistry is a broad subject matter. This review covered several aspects. It synthesizes current knowledge on soil pollution, environmental impacts, treatment technologies, and the critical role of soil chemical processes in contaminant fate. However, future reviews could focus on advanced characterization techniques such as in situ microscopy and spectroscopy; computational modeling of interfacial interactions; and hybrid remediation systems combining interfacial science with artificial intelligence-driven monitoring. Primarily, the novelty of this review lies in its interdisciplinary approach, combining interfacial chemistry with remediation science to provide dynamic, molecular-level insights. The review distinguishes itself from the existing literature by systematically linking interfacial chemical processes with practical remediation techniques. This approach provides a mechanistic understanding of pollutant behavior. Basically, this work contributes to the body of knowledge by examining contaminant behavior at heterogeneous interfaces. This information will assist in the optimization of adsorption, catalytic degradation, colloidal transport, and other cleanup strategies. Such molecular-level analysis is often missing in other reviews on soil pollution. Essentially, the integration of the knowledge of interfacial chemistry with remediation processes represents a crucial pathway toward more sustainable soil management in contaminated environments.

## 2. Significance of Soil Pollution

Soil pollution has emerged as one of the most pressing environmental challenges of our time, with far-reaching consequences that extend across ecological, human health, and socioeconomic domains. The contamination of soils with hazardous substances represents a critical threat to global sustainability, demanding immediate scientific attention and policy intervention [13]. The significance of soil pollution manifests through its impacts on ecosystem functioning, public health, agricultural productivity, and economic stability, creating complex challenges that require integrated solutions [14].

From an ecological perspective, polluted soils exhibit severe degradation of biological communities and ecosystem services. Contaminants fundamentally alter soil physicochemical properties, leading to the decline of microbial communities that are essential for nutrient cycling and organic matter decomposition [15]. Heavy metal contaminants such as Cd and Pb have been shown to disrupt critical enzyme activities and reduce microbial biomass, thereby compromising soil fertility and ecosystem productivity [16]. Polycyclic aromatic hydrocarbons and other organic contaminants show exceptional persistence in soil environments, preventing plant development and soil fauna populations while remaining biologically active for decades [17]. About 38% of soil pollution instances in Europe are caused by industrial and municipal waste, with the commercial and industrial sectors adding another 34% [18]. Nearly 60% of contamination episodes are caused by heavy metals and mineral oil, making them the most common pollutants in soil [18]. The European Environment Agency (EEA) estimates that between 250,000 and 340,000 contaminated sites need to be cleaned up throughout Europe, although there are likely many more [19].

The human health implications of soil pollution create equally grave concerns, with multiple exposure pathways facilitating the transfer of contaminants from soil to human populations. Direct contact through dermal absorption, inhalation of contaminated dust particles, and dietary intake via contaminated crops represent the primary routes of exposure that have been extensively documented in epidemiological studies [20,21]. The association between heavy metals in the soil and increased risks of various cancers, neurological disorders, and developmental abnormalities has been firmly established through longitudinal health studies [22,23]. For instance, in Bangladesh, widespread As contamination in rice-growing soils has created an unprecedented public health crisis affecting over 20 million people [23]. Furthermore, POPs such as dioxins and furans demonstrate concerning bioaccumulation potential in food chains, leading to well-documented cases of endocrine disruption and reproductive disorders in exposed populations [24,25].

Food security represents another critical dimension of the significance of soil pollution, with contamination directly threatening global agricultural productivity and food safety [26]. Soil pollution reduces agricultural productivity in heavily contaminated regions, creating substantial yield gaps that exacerbate global food insecurity [27,28]. Heavy metal accumulation in edible crops has reached alarming levels in certain regions, with Cd concentrations in rice exceeding safety limits by tenfold in some Chinese provinces [29]. The ability of soil pollutants to enter the human food chain creates long-term food safety concerns [30].

With respect to the impact of soil pollution on economic stability, soil pollution exacerbates public health expenditures, since exposure to contaminated soil leads to diseases such as lead poisoning, respiratory disorders, and cancers. Furthermore, soil pollution can cause a decline in land value and investment risks; as polluted land loses its economic value, consequently, discouraging investments in agriculture, real estate, and industrial development. Commonly, contaminated sites often remain abandoned due to high cleanup costs, leading to urban blight and reduced economic activity [31]. According to [31], bringing the Pb pollution of Ukrainian soils down to acceptable levels will enable the normative monetary value of arable land to rise 3.2% over the average level, freeing up funds for raising gross agricultural products. In Canada, both present and future generations of Canadian taxpayers have the financial responsibility of cleaning up federally polluted sites [32]. Global land degradation could result in losses of up to USD 23 trillion worldwide [33]. It is also worth mentioning that investors and businesses face financial risks when dealing with polluted land, as regulatory penalties and liability costs can be substantial.

To summarize how interfacial chemical processes align with the different perspectives of the impacts of soil pollution; from an ecological perspective, polluted soils exhibit severe degradation of biological communities and ecosystem services due to disrupted interfacial processes. This alters microbial adhesion and biofilm formation at soil particle interfaces, impairing nutrient cycling and organic matter decomposition. Comparably, agricultural productivity is severely compromised by soil pollution due to interfacial processes that affect plant uptake and soil fertility. Hydrophobic organic pollutants impede water and nutrient transport by clogging soil pores and coating particle surfaces, further reducing crop yields. Similarly, when pollutants become attached to soil particles, the value of the land typically decreases, discouraging investment because of the high costs and potential legal responsibilities associated with cleanup.

## 3. Historical Perspectives and Global Trends

Over the twentieth century, while legacy pollutants like Pb and dichlorodiphenyltrichloroethane (DDT) remained in the soil for decades, heavy metals, petroleum hydrocarbons, and synthetic chemicals were discharged into soils in extraordinary amounts [34]. The issue has taken on new dimensions in recent years due to the emergence of contaminants such as microplastics, pharmaceuticals, and per- and polyfluoroalkyl substances (PFAS) [35]. According to current estimates, pollution degrades a significant portion of the world’s soils, with hotspots found in fast industrializing countries like Asia and Africa [36,37]. Mitigation efforts are made more difficult by the transboundary character of soil contamination via food commerce, water runoff, and air deposition [38]. Even while human activity is the primary cause of soil pollution in recent times, organic compounds and trace metals are also released into terrestrial ecosystems by natural processes including forest fires, geological weathering, and volcanic emissions [39,40]. However, unless these natural processes are enhanced by human intervention, these natural inputs hardly ever reach dangerous levels, highlighting the necessity of focused pollution control measures.

Concerns about the accumulation of pesticides in the environment became prominent in the mid-20th century, prompting the introduction of major environmental regulations. The establishment of the Environmental Protection Agency (EPA) in 1970 and the passing of the Clean Air Act were key advances toward tackling pollution and chemical safety [41]. High-profile industrial disasters, such as the Love Canal crisis in 1978, brought national attention to the dangers of soil contamination, leading to the formation of the Superfund program in 1980 to remediate hazardous waste sites [42]. Internationally, the 2001 Stockholm Convention sought to eliminate or restrict POPs, which are toxic chemicals that can travel long distances and accumulate in ecosystems, affecting both human health and wildlife [43]. The European Union’s Soil Thematic Strategy of 2006 further strengthened efforts to prevent soil degradation across Europe [44]. In 2015, the United Nations adopted Sustainable Development Goal 15.3, which set a target to achieve land degradation neutrality by 2030, reinforcing the global commitment to sustainable soil and land management [45].

**Figure 1:**
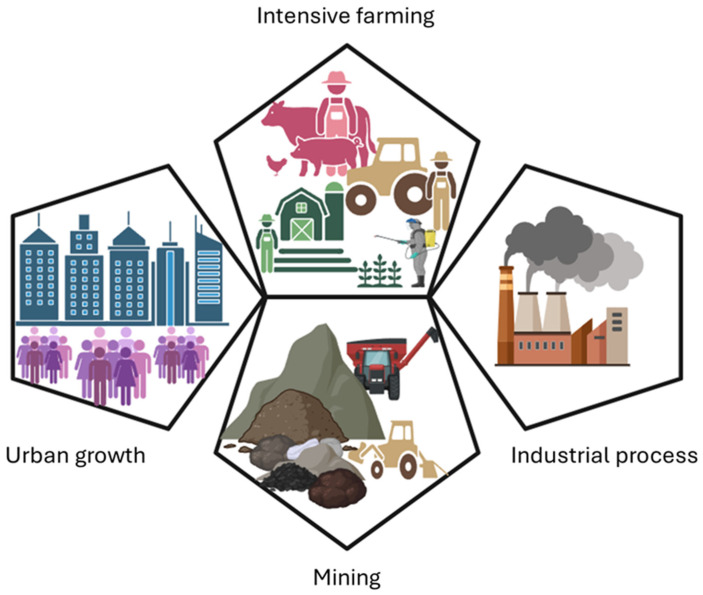
Sources of modern soil pollution.

Modern soil pollution stems from a wide range of human activities, such as intensive farming, industrial processes, urban growth, and mining (Figure 1).

**Figure 2:**
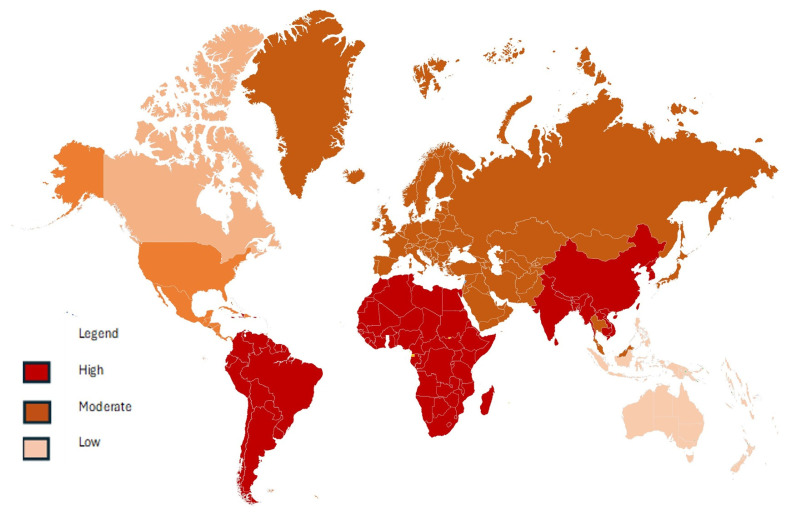
Global map showing heavy metal pollution levels (Adapted from [56,58,59]—Africa, Asia, and South America (high pollution index indicated by deep red color); Europe (mid pollution index represented as brown color) [50,51]; USA and Canada (moderate to low pollution index denoted by a mix of brown and light pink color) [54,55]; and Australia (low pollution index depicted by a light pink color) [57]).

Agriculture plays a major role by excessively using pesticides, fertilizers, and antibiotics, which can make soils toxic and less fertile. Industries contribute by releasing heavy metals like Pb, Cd, and As, as well as petroleum products and electronic waste, into the environment. Urbanization has exacerbated the problem through construction waste, and leachates from landfills, while mining produces acid mine drainage and causes toxic metals to seep into the soil. New types of pollutants are also emerging, further threatening soil health. For instance, microplastics from sewage sludge and discarded plastics accumulate in the soil and may disrupt microbial communities [46]. Pharmaceuticals, including antibiotics and hormones from animal farming and wastewater irrigation raise concerns about antibiotic resistance and hormonal imbalances [47]. Increasing amounts of engineered nanoparticles, such as titanium dioxide and silver, are also being found in soils, but their long-term effects are not yet fully understood. Furthermore, soil pollution varies by region (Figure 2). Specifically, in Asia, rapid industrialization in countries like China and India has caused significant heavy metal contamination; in Africa, artisanal mining and poor waste management contribute to soil degradation; and in Europe and North America, despite strict regulations, legacy pollution from past industrial activity and nuclear incidents remains a challenge [37,48,49]. The moderate level of heavy metal contamination observed in Europe is possibly due to strict environmental laws and consistent monitoring [50,51] (Figure 2). Between 2005 and 2022, the European Union reported reductions in emissions of Pb by 44%, mercury (Hg) by 53%, and Cd by 39% among its 27 member states [51].

Developed nations in North America and Europe showed lower levels of heavy metals than developing nations in Asia, Africa, and South America, following a study on 168 rivers and 71 lakes [52]. High food safety requirements are more likely to be met, and dangerous toxins are less likely to be present in fruits from areas with strict agricultural standards, like North America or Europe [53]. Specifically, in North America, contamination varies by region, with some areas facing moderate to low levels (Figure 2). In the North American regions, agricultural lands generally have lower metal concentrations, though Cu, nickel (Ni), and Pb may still exceed background thresholds [54,55]. In South America, particularly in Brazil, heavy metal accumulation is notably high in industrial zones compared to uncontaminated regions [56]. Meanwhile, Australia and its surrounding areas typically exhibit some of the lowest heavy metal contamination levels globally [57] (Figure 2).

In essence, the regional pollution patterns that have been discussed reveal stark contrasts. Developing regions in Asia and Africa face severe contamination due to rapid urbanization and loose regulations, where heavy metals and organic pollutants interact with soil colloids, altering their mobility and bioavailability. In contrast, developed zones like North America and Europe show lower contamination levels, attributed to stringent regulations that afford the minimization of the dynamics between soil pollutants and interfacial processes. However, even in these regions, localized hotspots exist where Cu, Ni, and Pb exceed background levels due to historical industrial activity. South America, particularly Brazil, demonstrates elevated heavy metal accumulation in industrial zones, possibly owing to redox conditions and organic matter content that govern pollutant speciation. Even though Australia shows low contaminant levels, anthropogenic pressures could disrupt this equilibrium.

## 4. Types and Characteristics of Selected Soil Pollutants

Soil contamination leads to the accumulation of dangerous substances that reduce soil fertility, hinder plant growth, and pose serious risks to human health [58,59]. The primary types of soil pollutants include inorganic substances like heavy metals [60], organic compounds such as petroleum hydrocarbons and PAHs [61,62], and pollutants of growing concern including pharmaceuticals [63], microplastics [46], and perfluorinated chemicals (PFCs) [64]. These pollutants enter the soil through multiple pathways: industrial waste discharges, agricultural chemical runoff, and improper disposal of waste materials [65,66]. Each category of contaminants behaves differently in the environment, affecting how long they persist, how they move through ecosystems, and their overall ecological impact [67]. While traditional pollutants like heavy metals and hydrocarbons have been studied extensively, emerging contaminants are now receiving increased attention due to their potential to impair soil health and disrupt ecosystems, even though research on them remains limited. This section will cover the different types of pollutants. It will also highlight modern methods for detecting and measuring contamination levels, which are crucial for developing effective soil treatment strategies [68].

### 4.1. Inorganic Pollutants (Heavy Metals)

Heavy metals are among the most persistent and hazardous inorganic pollutants in soils, posing significant risks to ecosystems, agricultural productivity, and human health [69]. Unlike organic pollutants, heavy metals are non-biodegradable and can accumulate in soil for decades, leading to long-term environmental damage [70]. The Earth’s crust naturally contains various heavy metals that are released into soil through weathering and the erosion of parent rocks. For example, As commonly occurs in sulfide minerals, while Cd is often associated with zinc (Zn) ores [39,71]. Volcanic eruptions and geothermal activities contribute to the atmospheric deposition of metals like Hg and Pb, which eventually settle into soils through precipitation [40,72]. Forest fires also release naturally occurring metals from vegetation into the air, leading to subsequent deposition over large areas [39]. These geogenic processes have influenced soil metal concentrations for ages, creating naturally metal-rich soils in some regions.

Conversely, human activities have dramatically accelerated the release of heavy metals into soils since the Industrial Revolution. Industrial operations such as mining, smelting, and metal processing are major point sources of contamination [73]. For instance, Pb and Zn smelters have created significant soil pollution hotspots worldwide [74,75]. The manufacturing sector contributes through improper disposal of metal-containing waste and effluents. Agricultural practices represent another significant pathway for metal accumulation in soils. The long-term application of phosphate fertilizers has introduced Cd into farmland soils, while copper (Cu)-based fungicides and As-containing pesticides have left lasting contamination [76,77]. Irrigation with wastewater or contaminated groundwater further exacerbates the problem in agricultural areas [78].

The persistence of heavy metals in soils is strongly influenced by adsorption and desorption mechanisms at mineral–organic interfaces. For instance, heavy metals like Cd, Pb, and As bind to soil components such as iron oxides, clay minerals, and organic matter through electrostatic interactions, ion exchange, or surface complexation [79]. These interfacial reactions determine whether metals remain immobilized or become bioavailable. Human activities, including industrial discharges and agricultural practices, can disrupt these equilibria by introducing competing ions or altering soil pH, thereby remobilizing metals into the environment. Dissolution and precipitation processes at mineral–water interfaces play a critical role in the natural release of heavy metals from geological sources [80]. Weathering of sulfide minerals, such as As-bearing rocks and Zn ores that are associated with Cd, release metals into soils, while subsequent interactions with soil constituents may lead to secondary mineral formation, such as metal phosphates or carbonates [81]. Agricultural inputs, like phosphate fertilizers, can induce interfacial precipitation reactions that temporarily immobilize metals, though changes in soil chemistry may eventually reverse these effects [82]. In essence, interfacial chemical processes provide the mechanistic foundation for the environmental persistence and risks of heavy metals.

### 4.2. Organic Pollutants (Petroleum Hydrocarbons and Polycyclic Aromatic Hydrocarbons)

Organic pollutants such as petroleum hydrocarbons and PAHs are persistent contaminants in soil systems, primarily originating from industrial discharges, accidental spills, and incomplete combustion of fossil fuels [83]. These compounds interact with soil matrices through interfacial chemical processes, including adsorption, partitioning, and hydrophobic interactions [84].

Petroleum hydrocarbons, composed of aliphatic and aromatic fractions, exhibit varying affinities for soil organic matter due to their hydrophobic nature [85]. The adsorption of petroleum hydrocarbons at the soil–water interface is governed by van der Waals forces and hydrophobic effects, where longer-chain hydrocarbons demonstrate stronger sorption [86]. Comparably, PAHs undergo π–π interactions with soil organic carbon, improving their retention [87]. The interfacial behavior of PAHs is further influenced by soil pH, clay mineralogy, and dissolved organic matter, which can either facilitate sequestration or promote mobilization [88]. Biodegradation of these pollutants is mediated by microbial activity at the soil–water interface, where bioavailability is a critical factor [89]. Additionally, surfactants and biosurfactants can enhance desorption by reducing interfacial tension, thereby increasing hydrocarbon accessibility to degrading microorganisms [90].

### 4.3. Emerging Pollutants (Pharmaceuticals, Microplastics, Perfluorinated Chemicals)

Emerging contaminants, including pharmaceuticals, microplastics, and PFCs, pose significant environmental risks due to their persistence and bioaccumulative potential [91]. Their fate in soil is largely dictated by interfacial interactions, including electrostatic forces, hydrogen bonding, and hydrophobic partitioning [92]. Pharmaceutical compounds, often polar and ionizable, interact with soil colloids through cation exchange, surface complexation, and hydrogen bonding [63]. Their mobility is influenced by soil pH and ionic strength, with cationic species exhibiting stronger adsorption to negatively charged clay minerals [35]. Microplastics, on the other hand, accumulate in soils through mechanical fragmentation and weathering, interacting with soil particles via hydrophobic and electrostatic forces [46]. Their large surface area facilitates the sorption of co-contaminants, acting as vectors for pollutant transport [93]. Perfluorinated chemicals exhibit unique interfacial behavior due to their amphiphilic nature [94]. Perfluorinated chemicals adsorb onto soil organic matter and mineral surfaces via hydrophobic and electrostatic interactions, with longer-chain compounds showing higher affinity [95].

In terms of their mechanism of soil contamination, animal dung and treated wastewater are the main ways that pharmaceuticals, such as hormones, antibiotics, and non-steroidal anti-inflammatory medications (NSAIDs), get into agricultural soils. These substances can linger, build up, and impact plant health and microbial populations once they are in the soil [96]. Depending on their chemical makeup, pharmaceuticals show varied levels of sorption to soil particles. For example, sulfonamides are more water soluble and more likely to leach, unlike antibiotics such as tetracyclines that bind to clay and organic matter strongly and reduce their mobility [97]. With regard to microplastics, they can change the structure of the soil. This impacts root growth by decreasing aeration and water retention [98]. While the strong carbon-fluorine linkages of PFCs help them to accumulate in soils [97,99]. Their long-term soil retention can result from their ability to withstand degradation [99]. They enter the food chain after being absorbed by plants and soil organisms [100]. Furthermore, PFCs pose broader environmental problems because of their surfactant qualities, which allow them to seep into groundwater [101]. Generally, sorption, leaching, and bioaccumulation are three different but related processes by which PFCs, microplastics, and pharmaceuticals pollute soil [99,101].

### 4.4. Instruments for Quantification of Selected Soil Pollutants

Accurate quantification of soil pollutants relies on advanced analytical techniques that exploit interfacial processes for extraction and detection. Gas chromatography-mass spectrometry (GC-MS) is commonly employed for the analysis of volatile and semi-volatile organics, such as petroleum hydrocarbons and PAHs [102]. Furthermore, interfacial interactions in the chromatographic column aid in compound separation [103]. High-performance liquid chromatography (HPLC) in conjunction with UV or fluorescence detection is used for polar emerging contaminants via solute-stationary phase interactions [104].

For microplastics, Fourier-transform infrared spectroscopy (FTIR) and Raman microscopy provide polymer identification by analyzing vibrational modes at particle surfaces [105]. Perfluorinated chemicals are quantified using liquid chromatography-tandem mass spectrometry (LC-MS/MS), where solid-phase extraction (SPE) enhances sensitivity by interfacial adsorption onto functionalized sorbents [106].

In addition, atomic force microscopy (AFM) and X-ray photoelectron spectroscopy (XPS) provide information on the molecular interactions between contaminants and adsorbents on their surfaces [107]. Other advanced techniques and instruments for heavy metal quantification in soil include Laser-Induced Breakdown Spectroscopy (LIBS), which rapidly detects heavy metals by analyzing plasma emissions generated from laser-ablated soil samples [108]. This technique requires minimal sample preparation and provides real-time results, making it suitable for industrial applications [109]. Inductively Coupled Plasma-Optical Emission Spectrometry (ICP-OES) is frequently employed for multi-element analysis due to its high sensitivity. It quantifies trace metals by exciting soil-digested samples in a plasma torch and measuring the emitted wavelengths [110]. Electrochemical sensors, including anodic stripping voltammetry (ASV), enable on-site detection of heavy metals such as Hg and Cu through redox reactions at modified electrode surfaces [111]. Synchrotron-Based X-ray Absorption Spectroscopy (XAS) provides detailed insights into metal speciation and bonding at the atomic level, and it is particularly useful for assessing the bioavailability of heavy metals in contaminated soils [112]. Micro-X-ray Fluorescence (μ-XRF) allows for high-resolution mapping of metal distribution within soil matrices, aiding in the assessment of localized contamination hotspots [113]. These techniques underscore the role of interfacial chemistry in pollutant detection, enabling precise environmental monitoring and risk assessment [114].

## 5. Relationship Between Soil Treatment and Interfacial Chemistry

The study of soil pollution is intrinsically linked to interfacial chemistry, which explores the chemical interactions occurring at the boundaries between soil particles, water, air, and contaminants. These interactions dictate the mobility, persistence, and toxicity of pollutants, making interfacial chemistry a crucial field for developing effective soil remediation strategies. The fate, transport, and bioavailability of contaminants are determined by these interactions. Important processes include adsorption and desorption, in which pollutants attach to clay minerals or soil organic matter and are impacted by surface charge, ionic strength, and pH [115]. Under reducing conditions, redox processes are important for transforming pollutants like chromium (Cr (VI)) into less harmful forms such as Cr (III). In addition, complexation is the process by which metals and ligands, including humic acids, generate soluble or insoluble complexes [116]. For example, the mobility of Cd in soils is strongly influenced by pH, with acidic environments encouraging its movement into groundwater [117].

Similarly, hydrophobic organic compounds like PAHs preferentially sorb to soil organic carbon, limiting their biodegradation [118]. Despite advances in remediation technologies, ranging from phytoremediation to nanotechnology, the effectiveness of soil treatment methods hinges on a nuanced understanding of interfacial chemistry [90]. For example, electrokinetic remediation relies on manipulating soil pH and electric gradients to mobilize heavy metals [118], while microbial degradation of PAHs is enhanced by surfactants that increase contaminant bioavailability [90]. However, gaps remain in translating laboratory-scale findings to field applications, particularly for heterogeneous soil systems [119].

### 5.1. Interfacial Chemistry Drivers

The interfacial chemistry principle provides essential insights for several critical aspects of soil pollution management. Precisely, it explains the fundamental mechanisms by which contaminants are retained or released from soil matrices. Furthermore, it predicts the potential for contaminant migration through soil profiles and into groundwater systems. Also, it determines the long-term stability of contaminants in soil environments. In addition, and most importantly for remediation purposes, it governs the effectiveness of various treatment technologies by controlling the accessibility of contaminants to biological, chemical, or physical treatment processes.

#### 5.1.1. Soil–Contaminant Interactions

Soil mineralogy plays a significant role, with clay minerals (smectites, kaolinites) providing extensive surface areas for contaminant binding, through mechanisms such as ion exchange and surface complexation [120]. Iron and manganese oxides represent reactive soil components that strongly interact with both metallic and organic contaminants through surface precipitation and redox reactions [121]. Soil organic matter constitutes another crucial component influencing contaminant behavior. The humic and fulvic acid fractions provide numerous functional groups (carboxyl, phenolic, carbonyl) that participate in complexation reactions with metal ions and hydrophobic interactions with organic contaminants [122]. The surface charge characteristics of soil particles, as quantified by zeta potential measurements, significantly influence contaminant interactions [123]. Most soil particles carry a net negative charge under typical pH conditions, leading to strong electrostatic interactions with cationic contaminants, for example, heavy metals, but causes repulsion of anionic species (arsenate and chromate). The point of zero charge, where the net surface charge is neutral, represents a critical threshold that can dramatically alter contaminant adsorption behavior [123].

#### 5.1.2. Adsorption Mechanisms

Adsorption processes at soil interfaces can be conceptually divided into several distinct mechanisms, each with different implications for contaminant retention and remediation potential. Physical adsorption (physisorption) involves relatively weak van der Waals forces and typically results in reversible binding of contaminants to soil surfaces [124]. The sorption mechanism predominates nonpolar organic compounds interacting with soil organic matter, following the principles of hydrophobic partitioning [88].

Chemical adsorption (chemisorption) entails much stronger interactions, often involving covalent bonding or ionic interactions between contaminant species and specific surface functional groups [88,124]. For metal ions, this frequently takes the form of inner-sphere surface complexes where water molecules are excluded from the coordination sphere of the surface-bound metal [125]. Such chemisorption processes are less irreversible and may require aggressive chemical treatments for contaminant release [126,127].

Ion exchange represents a particularly important adsorption mechanism in soils with significant clay content. The negative charges on clay mineral surfaces attract exchangeable cations such as calcium, sodium, and potassium ions (Ca^2+^, Na^+^, K^+^), which can be displaced by contaminant metal ions according to mass action principles [128].

Surface precipitation occurs when contaminant concentrations exceed solubility limits at the particle surface, leading to the formation of insoluble contaminant-bearing solid phases [129]. This mechanism is particularly relevant for metal contaminants under alkaline conditions, where metal hydroxides or carbonates may precipitate at particle surfaces [130]. While this represents an effective immobilization mechanism, such precipitates may re-dissolve under changing environmental conditions [130].

#### 5.1.3. Mobility Modifiers

The mobility of contaminants in soil systems is controlled by numerous environmental factors. Soil pH is one of the important factors that influences both the surface charge properties of soil particles and the speciation of pollutants [131]. For metal contaminants, lower pH conditions generally increase solubility and mobility due to proton competition for surface sites and dissolution of metal-bearing phases [75]. Conversely, higher pH conditions typically favor metal adsorption and precipitation, though some oxyanions show increased mobility at alkaline pH [75,132].

Redox potential represents another crucial factor governing contaminant mobility, particularly for elements capable of existing in multiple oxidation states (e.g., Fe, Mn, Cr, As) [133]. Reducing conditions may promote the reductive dissolution of iron and manganese oxides, releasing associated contaminants into the solution [121]. Alternatively, reducing conditions may immobilize certain contaminants through reductive precipitation, for example, the reduction of Cr (VI) to Cr (III)) [134].

The presence of dissolved organic matter can significantly enhance contaminant mobility through complexation reactions [135]. Dissolved organic matter contains numerous functional groups capable of forming soluble complexes with metal ions, effectively competing with soil surfaces for contaminant binding [136]. For hydrophobic organic contaminants, dissolved organic matter may act as a carrier phase, increasing apparent solubility through a process analogous to co-solvency [135].

The ionic strength of soil solution influences contaminant mobility through its effects on electrostatic interactions. Higher ionic strength compresses the electrical double layer around charged soil particles, potentially reducing the effective range of electrostatic attraction for oppositely charged contaminants [137,138].

#### 5.1.4. Contaminant Aging Effects

The phenomenon of contaminant aging refers to the progressive changes in contaminant–soil interactions over time, generally leading to decreased contaminant bioavailability and extractability [139,140]. These changes occur through multiple mechanisms operating at different time scales. Short-term aging (days to weeks) may involve diffusion into soil micropores or structural defects in mineral particles [141]. Medium-term processes (months to years) may include incorporation into newly forming mineral phases or stronger chemical bonding with soil organic matter. Over longer time scales (years to decades), contaminants may become occluded within mineral structures or transformed into more stable chemical forms [141]. Generally, aged contaminants exhibit decreased bioavailability to microorganisms, which limits biodegradation [142]. The aged contaminants also exhibit decreased chemical extractability, therefore hindering chemical treatments [139,140]. This aging effect necessitates more aggressive remediation approaches for historically contaminated soils compared to freshly contaminated sites [119].

#### 5.1.5. Molecular-Scale Dynamics Phenomena

Molecular-scale dynamics phenomena at soil interfaces play a critical role in determining contaminant mobility within natural porous media [143]. Recent advances in experimental and computational techniques have revealed that slow relaxation processes, dynamic heterogeneity, and confinement effects collectively govern interfacial reactions and transport mechanisms, challenging traditional homogeneous models of soil chemistry [144].

- Slow Relaxation and Time-Dependent Transport Dynamics

Interfacial reactions in soils often exhibit non-equilibrium behavior due to slow relaxation processes [145]. Pulsating flow conditions induce delayed contaminant release by altering colloid mobilization and pore-scale stress distributions [146]. For example, during drying stages, when capillary forces break pore walls and produce colloids loaded with pollutants, polybrominated diphenyl ethers (PBDEs) at e-waste sites exhibit hysteresis in release kinetics [147]. Similarly, temporal moment analyses in low-permeability porous media reveal asymmetric contaminant plume evolution, with mean residence times extending significantly in heterogeneous systems due to kinetic limitations in sorption-desorption equilibria [148]. These findings show the limitations of steady-state models in forecasting the long-term behavior of contaminants.

- Dynamic Heterogeneity at Molecular Interfaces

Soil interfaces are fundamentally heterogeneous, with reactive sites varying in composition, topology, and local coordination environments [149]. For example, molecular dynamics simulations of film water at silica–water interfaces demonstrate how hydroxyl group density creates spatially varying diffusion coefficients, leading to orders-of-magnitude differences in contaminant mobility between adjacent surface regions [150]. Furthermore, studies show that convection dominates contaminant transport at soil-perched water interfaces [151,152], while diffusion governs migration in pore water systems, with some forest models identifying threshold effects in hydraulic conductivity that abruptly accelerate pollutant breakthrough [151].

- Confinement Effects

In terms of confinement effects in nanostructure environments, spatial confinement within soil nanopores amplifies interfacial forces, consequently, altering contaminant behavior [153]. For example, hydroxylated silica surfaces greatly reduce the diffusion rates of heavy metals in comparison to bulk water by promoting the formation of solid-like water through hydrogen bonding [154]. These nanoconfined environments also promote unusual reaction pathways, delaying redox transformations [155]. Permeable reactive barriers leveraging clay-rich soils exploit these confinement effects, achieving greater retention of cationic dyes through synergistic electrostatic trapping and pore constriction mechanisms [156,157]. However, confinement-induced aggregation of hydrophobic contaminants can enhance colloid-facilitated transport during pulsating flows, making remediation efforts challenging [157].

- Pollutant Retention at Soil Interfaces

The complex behavior of contaminants in soil systems exhibits striking parallels with dynamic phenomena observed in soft condensed matter physics, particularly regarding slow relaxation processes under confinement and disorder [158]. Recent advances in interfacial soil chemistry have revealed that classical equilibrium models fail to capture the time-dependent retention and remobilization of pollutants, necessitating new theoretical frameworks drawn from polymer physics and glassy dynamics [159,160]. The work by [161] in semi dilute polymer solutions under confinement provides critical insights into the anomalous transport of contaminants in soil microstructures. Their experimental demonstration of stretched exponential relaxation kinetics, described by the Kohlrausch–Williams–Watts function [162], directly informs the understanding of organic pollutant release from soil organic matter. Similarly, ref. [163] explored how PAHs and humic molecules, specifically, glomalin-related soil protein (GRSP) interacts, revealing GRSP’s role in binding PAHs and influencing the transport and transformation of organic pollutants in soil. In another study by [164], the authors established that structural disorder in colloidal glasses leads to spatially and temporally intermittent relaxation events, a phenomenon that is presently recognized in soil systems through advanced microscopy techniques. Recent work by [165] has demonstrated similar dynamic heterogeneity in the migration of PFAS through organic matter matrices. These observations fundamentally challenge traditional adsorption–desorption models by revealing the microscale origins of macroscopic pollutant retention.

## 6. Soil Treatment Technologies

**Figure 3:**
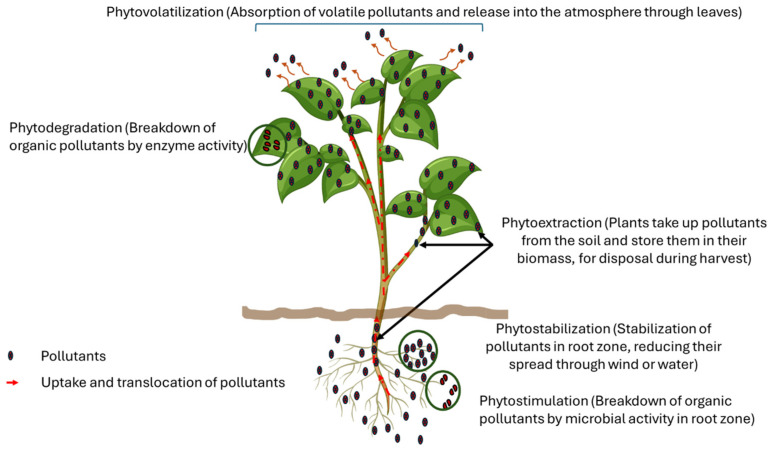
Phytoremediation techniques.

Interactions such as adsorption, ion exchange, and surface complexation influence whether pollutants remain bound to soil particles or leach into groundwater. Specifically, heavy metals often form strong complexes with organic matter and clay minerals, reducing their immediate toxicity by immobilizing them. Essentially, remediation approaches such as phytoremediation, electrokinetic treatment, and soil washing rely on altering contaminant–soil interactions through physical, chemical, or biological means. Specifically, phytoremediation techniques including phytovolatilization, phytodegradation, phytoextraction, phytostabilization, and phytostimulation utilize interfacial processes to either break down pollutants or separate them from the soil matrix [166] (Figure 3). Technically, interfacial processes underpin modern soil remediation techniques. Addressing soil contamination requires tailored solutions that account for pollutant type, site characteristics, and risk thresholds. Some of the major soil treatment techniques are discussed herein.

### 6.1. Biological Treatment

Biological methods rely on interfacial processes to make contaminants available for microbial degradation. In bioremediation, microorganisms must either directly make a contact with adsorbed contaminants or rely on their desorption into the aqueous phase, while phytoremediation leverages plant–soil interactions to extract or stabilize contaminants [167] (Figure 3).

### 6.2. Chemical Treatment

Chemical remediation approaches directly manipulate interfacial chemistry to immobilize or extract contaminants. These include surfactant inclusion, catalytic irradiation, soil washing [168], soil vapor extraction, and advanced oxidation processes (AOPs) [119]. These AOPs include photocatalytic degradation and in situ chemical applications (ISCAs). These ISCAs entail the introduction of chemical oxidants into subterranean soil [119]. This enables the removal of pollutants in soil matrices. For example, phosphate compounds can promote pyromorphite formation with Pb, while iron oxides can provide new adsorption sites for As [169]. Soil washing is another chemical treatment procedure that uses chemical extractants to break the bonds between contaminants and their surfaces. The selection of extractants (acids, chelators, surfactants, and others) depends on the specific chemistry of the contaminants [168]. In addition, acid extraction works well for metals bound by pH-sensitive mechanisms, while chelators target specifically coordinated metals [170].

### 6.3. Physicochemical Treatment

Adsorption technique is one of the predominantly applied physicochemical methods for soil treatment. Another example is the stabilization/solidification soil treatment. This is also physicochemical in nature. This technique involves the formation of new mineral phases that encapsulate contaminants [171]. The formation of calcium silicate hydrate phases in cement-based treatments can incorporate metals into their structure [172]. The long-term stability of these phases depends on the prevalent environmental condition [171,172].

**Figure 4:**
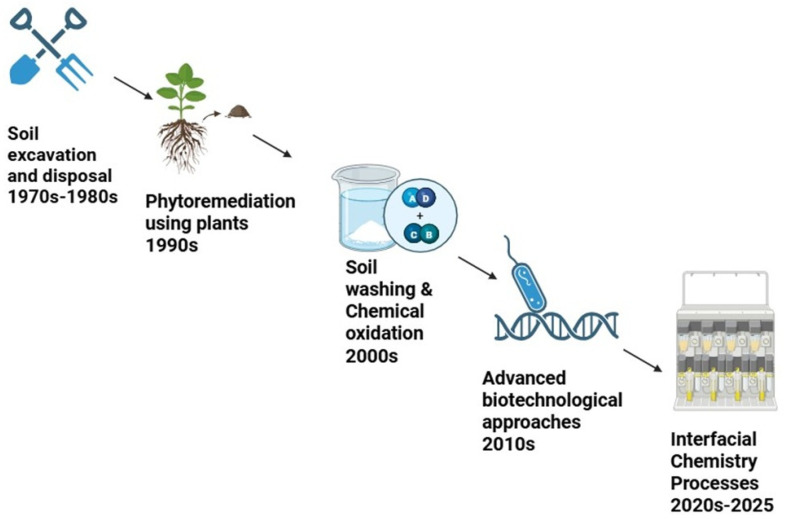
Key advancements in remediation methods.

Most importantly, to clarify the different mechanical aspects, biological treatment relies on interfacial processes that involve the microbial degradation of contaminants either through direct contact or via biosurfactant-mediated solubilization, as seen in *Geobacter* species facilitating reductive dechlorination [173], while phytoremediation leverages plant–soil interactions, with hyperaccumulators that can extract heavy metals through root exudates and metal transporters [166]. Conversely, chemical methods manipulate interfacial chemistry, such as AOPs, generating OH radicals to break down pollutants via photocatalysis or persulfate activation [119]. Comparably, soil washing employs acids, chelators, or surfactants to disrupt metal–soil bonds, with reagents such as ethylenediaminetetracetic acid (EDTA) effectively complexing metal ions for extraction [168]. Stabilization methods such as phosphate-induced pyromorphite production immobilize metals by changing their speciation [171]. Furthermore, physicochemical adsorption utilizes materials like biochar, whereby electrostatic and hydrophobic interactions help to trap contaminants on binding sites on adsorptive surfaces [126]. These technologies collectively address contaminant mobility, bioavailability, and degradation, offering tailored solutions for soil restoration. Figure 4 shows the progressive sophistication of remediation technologies from the 1970s to 2025.

## 7. Soil Remediation Investments

**Figure 5:**
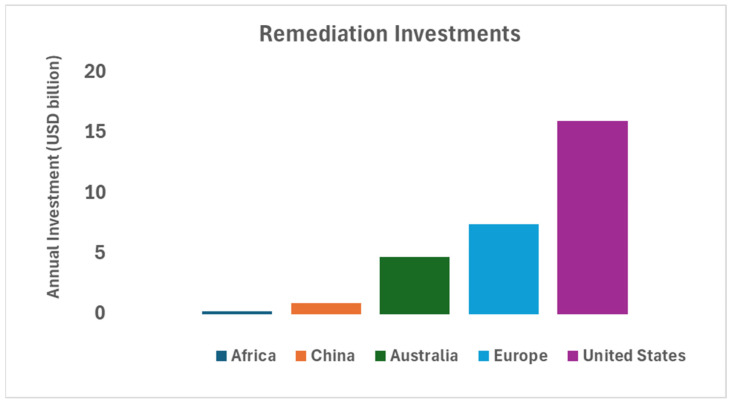
Annual remediation investments by major economies (Adapted from [174,175,176,177,178,179]).

During the 26th United Nations Climate Change Conference (COP26), in November 2021, a project was launched to raise USD 2 billion to restore 100 million hectares of damaged land in Africa, from 2022 to 2030 [174,175]. As part of China’s 12th Five-Year Remediation Plan (2011–2015), about USD 4.55 billion was allocated for soil cleanup efforts, with a focus on urban regions [176]. For 2022, the United States designated roughly USD 15.96 billion through the Bipartisan Infrastructure Law to restore abandoned mines and address orphaned well sites [177]. The annual cost of managing polluted land in Europe is around USD 7.43 billion [178]. Following research and studies, 24 interventions worth about USD 4.73 billion per year have been suggested for the remediation of degraded soil in Australia [179]. Figure 5 shows a bar chart of the planned/completed annual remediation investments of the different economies using calculated estimates from these published data.

## 8. Future Directions

To combat soil pollution, there is a need for the application of effective solutions. Key areas could include elucidating contaminant binding mechanisms using advanced spectroscopy and simulations, particularly for heavy metals and organic pollutants like PFAS and PAHs. Testable hypotheses could include the examination of how specific functional groups in organic matter influence the adsorption affinity of PFAS compounds in different soil types and an assessment to ascertain if competitive ion exchange dominates over surface complexation in heavy metal retention under varying pH conditions. Pollutant immobilization and phytoremediation can be enhanced by knowledge of rhizosphere chemistry and redox-driven processes. Research in this area could include an investigation on how root exudates from hyperaccumulator plants alter the speciation and bioavailability of metal ions in contaminated soils. Another study could also be centered on improving the extraction efficiency of metal ions in anaerobic soils via electrokinetic-assisted phytoremediation. Nanomaterial–soil interactions, such as engineered nanoparticles for targeted degradation, and micro-plastic-pollutant co-transport require further study. Concrete research questions might include an assessment of the long-term stability and unintended ecological impacts of iron oxide nanoparticles used for in situ PAH degradation. Also, researchers could conduct more studies that will determine if microplastics act as vectors for increased leaching of hydrophobic pesticides in sandy soils. Emerging contaminants, including antibiotic resistance genes and short-chain PFAS, require investigation into their interfacial behavior. It will be useful for researchers to find out if short-chain PFAS are more prone to leaching in agricultural soils compared to long-chain variants due to weaker sorption. Climate change effects, such as wetting–drying cycles on contaminant release, must also be explored to design resilient remediation approaches. Bridging lab-scale findings to field applications through in situ techniques and pilot testing will ensure practical solutions. Ultimately, integrating interfacial chemistry with soil science, engineering, and policy will enable innovative and scalable pollution mitigation strategies.

## 9. Conclusions

This review illustrates the interrelation between interfacial chemistry and soil pollution by shedding light on how interfacial processes determine the fate, mobility, and remediation of contaminants in soil. Pollutants like heavy metals and organic compounds bind to soil particles such as clays and organic matter through surface reactions. Interfacial chemistry helps to optimize adsorption and immobilization, thereby reducing pollutant mobility. Also, at interfaces, chemical transformations such as catalytic surfaces or reactive minerals can degrade organic pollutants via redox reactions. In the area of colloid transport, nanoparticles and colloids can carry pollutants through soil, and interfacial chemistry helps to stabilize or destabilize the colloids, consequently, enhancing soil remediation. In addition, surfactants modify interfacial tension, subsequently improving the removal of hydrophobic pollutants during soil washing. To mitigate soil contamination, sustainable agricultural practices and efficient remediation strategies need to be implemented. Cost-effective adsorbents such as biochar, activated carbon, and clay minerals can be utilized to immobilize heavy metals and organic pollutants. To enhance contaminant binding, surfaces of adsorbents can be modified with functional groups like carboxyl or amine groups. Non-hazardous but effective oxidation processes like Fenton’s reagent and zero-valent iron (ZVI) can be applied to degrade organic pollutants. The multidisciplinary approach of linking soil remediation with interfacial chemistry distinguishes the present report from other soil pollution reviews. The review provides relevant molecular-level insights that are crucial for the treatment of polluted soil. Details from this review could be important to a diverse group of people, including soil scientists, researchers, environmental scientists, chemical engineers, ecologists, environmental microbiologists, policy makers, industry, and government agencies. Overall, the incorporation of interfacial chemistry and its dynamics in soil treatment approaches will help to improve the efficiency of remediation technologies.
